# Maternal Secondhand Smoke Exposure Enhances Macrosomia Risk Among Pregnant Women Exposed to PM_2.5_: A New Interaction of Two Air Pollutants in a Nationwide Cohort

**DOI:** 10.3389/fpubh.2021.735699

**Published:** 2021-11-18

**Authors:** Yunyun Luo, Yuelun Zhang, Hui Pan, Shi Chen

**Affiliations:** ^1^Key Laboratory of Endocrinology of National Health Commission, Department of Endocrinology, State Key Laboratory of Complex Severe and Rare Diseases, Peking Union Medical College Hospital, Chinese Academy of Medical Science, Peking Union Medical College, Beijing, China; ^2^Medical Research Center, Peking Union Medical College Hospital, Chinese Academy of Medical Sciences and Peking Union Medical College, Beijing, China

**Keywords:** fine particulate matter (PM_2.5_), interaction, birth weight, air pollution, secondhand smoking

## Abstract

**Background:** Fine particulate matter (PM_2.5_) is one of the most common outdoor air pollutants, and secondhand smoking (SHS) is an important source of inhalable indoor air pollution. Previous studies were controversial and inconsistent about PM_2.5_ and SHS air pollutants on neonatal birth weight outcomes, and no studies assessed the potential interactive effects between PM_2.5_ and SHS on birth weight outcomes.

**Purpose:** To investigate the interaction between gestational PM_2.5_ and SHS air pollution exposure on the risk of macrosomia among pregnant women and examine the modifying effect of SHS exposure on the association of PM_2.5_ air pollution and birth weight outcomes during pregnancy.

**Methods:** Research data were derived from the National Free Preconception Health Examination Project (NFPHEP), which lasted 3 years from January 1, 2010, to December 31, 2012. At least 240,000 Chinese women from 220 counties were enrolled in this project. PM_2.5_ exposure concentration was obtained using a hindcast model specific for historical PM_2.5_ estimation from satellite-retrieved aerosol optic depth. Different interaction models about air pollution exposure on birth weight outcomes were established, according to the adjustment of different confounding factors and different pregnancy stages. The establishment of interaction models was based on multivariable logistic regression, and the main confounding factors were maternal age at delivery and pre-pregnancy body mass index (BMI) of participants. SHS subgroups analysis was conducted to further confirm the results of interaction models.

**Results:** In total, 197,877 participants were included in our study. In the full-adjusted interaction model, maternal exposure to PM_2.5_ was associated with an increased risk of macrosomia in whole, the first-, second-, and third trimesters of pregnancy (*p* < 0.001). The interactive effect was statistically significant between maternal exposure to PM_2.5_ and SHS on the risk of macrosomia in the whole (interaction *p* < 0.050) and the first-trimester pregnancy (interaction *p* < 0.050), not in the second (interaction *p* > 0.050) or third trimester (interaction *p* > 0.050) of pregnancy. The higher frequency of SHS exposure prompted the stronger interaction between the two air pollutants in the whole pregnancy and the first-trimester pregnancy.

**Conclusions:** In the whole and first-trimester pregnancy, maternal exposure to SHS during pregnancy enhanced the risk of macrosomia among pregnant women exposed to PM_2.5_ air pollutants, and the interaction became stronger with the higher frequency of SHS exposure.

## Introduction

Birth weight is a key indicator in newborn and infant survival and is associated with health outcomes across the whole life course, including cardio-metabolic and mental health, some cancers, and mortality (1, 2). A fetus larger than 4,000 g is considered macrosomia, regardless of gestational age (3). Macrosomia is associated with an increased risk of maternal and fetal complications, such as birth canal trauma, shoulder dystocia, and perinatal asphyxia (4). Compared with infants born at normal weights, macrosomia newborns are at more risk for long-term complications, such as obesity and insulin resistance (5). It was well-established that both genetic and environmental elements played indispensable roles in pregnancy outcomes, such as birth weight (6, 7). Genetic elements include parental hereditary material, epigenetic variation, and maternal placenta factors (8, 9), while environmental elements include gestational air pollutants, maternal addiction to smoking or alcohol, and maternal nutritional conditions (10, 11). It is more practicable to control environmental elements than genetic elements to improve adverse pregnancy outcomes. Among environmental elements, fine particulate matter (PM_2.5_) is one of the most common outdoor air pollutants caused by fossil fuel burning and vehicle emissions, while secondhand smoking (SHS) is an important source of inhalable indoor air pollution produced by smokers (12, 13). Both air pollutants are mainly caused by human activities and are likely to be changed and avoided by taking some measures. Pregnant women exposed to PM_2.5_ during pregnancy can lead to adverse pregnancy outcomes, such as fetal congenital malformation, premature delivery, abnormal birth weight of the fetus, and in severe cases, neonatal death (14, 15). Gestational SHS exposure at work and/or at home increased the risk of fetal stillbirth, neonatal death, and perinatal death (16).

Previous studies have established strong epidemiological linkages between pre-natal exposure to PM_2.5_ or SHS and fetal birth weight outcomes. Concerning PM_2.5_, the majority of studies reported that the increased risk of low birth weight (LBW) was associated with maternal exposure to PM_2.5_ (17). Links between gestational PM_2.5_ exposure and small for gestational age (SGA) have been observed in some studies (10). A nationwide cohort study also suggested that gestational exposure to PM_2.5_ during pregnancy was significantly associated with an increased risk of macrosomia (18). In terms of SHS, evidence on the association of neonatal birth weight and gestational cigarette smoke exposure was accumulated with a growing number of studies (7, 19, 20). The LBW prevalence was higher among newborns whose mothers were pre-natally exposed to SHS comparing with newborns whose mothers were not exposed (7). The link between maternal passive smoking and increased risk of delivering SGA also exists (20). All in all, the findings of the above studies about PM_2.5_ and SHS air pollutants on neonatal birth weight outcomes have been controversial and inconsistent.

There are some research gaps in the areas of association between environmental pollutants and newborn birth weight. First, whether the PM_2.5_ pollutants and SHS were associated with an increase or decrease in birth weight was unclear. Second, most related studies were confined to certain cities or provinces. The research based on nationwide cohorts was limited and scarce. Third, all the previous studies have separately evaluated the effects of PM_2.5_ or SHS air pollutants on birth weight outcomes, or considered SHS as a possible confounding factor in studies. No published studies have assessed the potential interaction between PM_2.5_ and SHS and evaluated the interactive effects on birth weight outcomes.

In our study, we aimed to investigate the interaction between gestational PM_2.5_ and SHS air pollution exposure on the risk of macrosomia among pregnant women and examine the modifying effect of SHS exposure on the association of PM_2.5_ air pollution and birth weight outcomes during pregnancy. We conducted the data cleaning process following strict screening standards to ensure the inclusion eligibility of all the enrolled women. We established different interaction models about air pollution exposure on birth weight outcomes, according to the adjustments of different confounding factors, and different pregnancy stages. SHS subgroups analysis was conducted to further confirm the results of interaction models. Our study expanded the region of investigation, achieving nationwide evidence, including 220 countries from 31 provinces or municipalities in China. This study was the first nationwide, population-based prospective cohort study to identify the interactions between gestational PM_2.5_ and SHS exposure among pregnant women on birth weight outcomes.

## Methods

### Study Design and Participants

**Figure 1:**
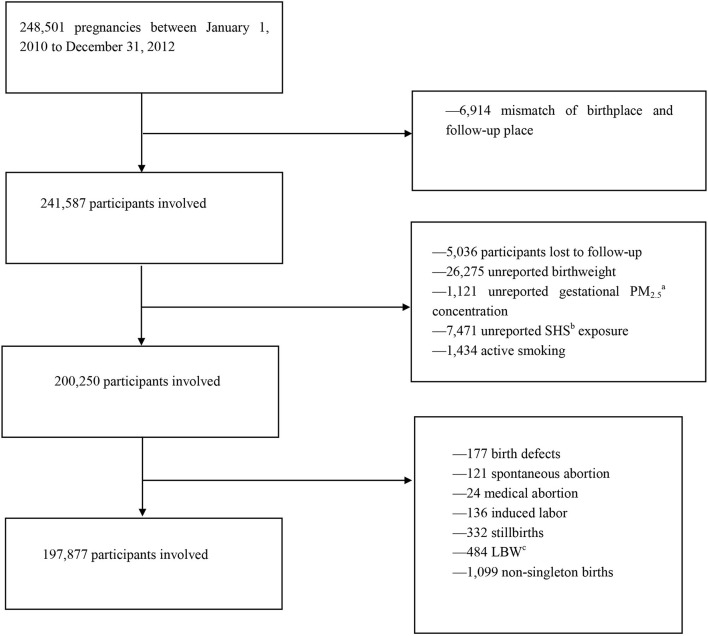
Flowchart of participants' inclusion and exclusion. ^a^PM_2.5_, fine particulate matter. ^b^SHS, secondhand smoking. ^c^LBW, low birth weight.

Our research data were derived from the National Free Preconception Health Examination Project (NFPHEP), which has lasted 3 years from January 1, 2010, to December 31, 2012. This project was launched by the National Health and Family Planning Commission and the Ministry of Finance of the People's Republic of China in 2010. The NFPHEP aimed to provide free preconception health examinations in rural areas to married couples who planned to get pregnant within the next 4–6 months. Well-trained local community health workers and obstetricians provided free preconception counseling, medical examinations, and management during pregnancy. In all, at least 240,000 Chinese women in 220 counties from 31 provinces or municipalities of China were enrolled in this project. In this study, we initially obtained 248,501 pregnant women from the NFPHEP. The follow-up success rate of the study is about 97.97% according to the dataset. After 6,914 mismatches of birthplace and follow-up place or missing were removed, 241,587 participants were involved preliminarily. Participants lost to follow-up (5,036), unreported birth weight (26,275), unreported gestational PM_2.5_ concentration (1,121), unreported SHS exposure (7,471), and active smoking (1,434) were excluded. Pregnant women with adverse pregnancy outcomes, including birth defects (177), spontaneous abortion (121), medical abortion (24), induced labor (136), stillbirths (332), non-singleton births pregnancies (1,099), and LBW (484), were also excluded from the study. The selection process, including reasons for excluding some pregnant women, is summarized in a flow diagram (Figure 1). Finally, 197,877 participants were included in the current study. In addition, multiple imputations were also done for missing data, including the concentration of PM_2.5_ in different pregnancies, birth weight, SHS conditions, and the flowchart for the inclusion and exclusion of participants is added in Supplementary Figure A.1.

The study was approved by the institutional review board of the National Research Institute for Family Planning, Beijing, China. All participants provided written informed consent. The detailed design and implementation of NFPHEP have been described elsewhere (18, 21, 22).

### Referred Variables

In this project, information of both enrolled pregnant women and neonates were collected by face-to-face investigation and examination conducted by trained and qualified staff. In total, 371 items in 19 aspects, including social demographic characteristics, family history, lifestyle factors, history of diseases, physical examinations, laboratory tests, and diagnostic imaging, were obtained. All data were uploaded to a nationwide electronic data capture system and underwent quality control by the National Quality Inspection Center for Family Planning Techniques.

The variables analyzed in our study included PM_2.5_ concentration, age at delivery, birth weight of neonate, sex of neonate, educational level, smoking during pregnancy, alcohol intake during pregnancy, prolonged pregnancy, multiparity, pre-pregnancy BMI, pre-pregnancy diabetes mellitus, pre-pregnancy hypertension, family history of diabetes mellitus, and the season of delivery.

### Outcome and Exposure Assessment

As recommended by the WHO, a newborn baby ≥4,000 g is considered macrosomia (23). We defined normal birth weight (NBW) as the birth weight between 2,500 and 4,000 g (23). The weight of each newborn was recorded by information recorders. In this study, the PM_2.5_ exposure concentration of each country was provided by the Chinese Center for Disease Control and Prevention through a hindcast model specific for historical PM_2.5_ estimation from satellite-retrieved aerosol optic depth. It was an ensemble machine-learning model that provided reliable PM_2.5_ hindcast capabilities comprised of random forest, generalized additive model, and extreme gradient boosting. The missing satellite data were first filled by multiple imputations. To control for unobserved spatial heterogeneity, China was divided into seven regions through a spatial clustering method. Ensemble machine-learning models above were trained in each region separately. Satellite data and PM_2.5_ ground monitoring records at 1,593 monitoring stations across mainland China from 2013 to 2016 were used to train the model. Finally, a generalized additive ensemble model was developed to combine predictions from different algorithms. The ensemble prediction characterized the spatiotemporal distribution of daily PM_2.5_ well with the cross-validation (CV) *R*^2^ (root mean square error, RMSE) of 0.79 (21 μg/m^3^). The cluster-based sub-region models outperformed national models and improved the CV R^2^ by ~0.05. Specific parameter settings and model building have been reported in detail elsewhere (18, 24). Daily county-specific PM_2.5_ concentration of each included pregnant woman was used to calculate monthly PM_2.5_ concentration to evaluate PM_2.5_ exposure condition during the pregnancy in our study. The average monthly PM_2.5_ concentrations in 1–3 months' gestation, 4–6 months' gestation, and 7 months to delivery were regarded as the first-, second-, and the third- trimester of pregnancy, respectively. Address information of each woman at the county level has been registered. SHS is passive inhalation of cigarette smoke produced by active smoking of smokers, also commonly known as “passive smoking” or “forced smoking.” Women in our study may be exposed to different degrees of SHS in the workplace or at home. They were divided into three groups according to exposure levels. Being exposed to one full cigarette was defined as one time. Those who never touched smokers or secondhand smokers were classified into the “None exposure SHS” subgroup. Then being exposed to smoking 1–6 times per week was defined as “Occasional Secondhand Smoke Exposure,” and being exposed to smoking ≥ 7 times per week was defined as “Frequent Secondhand Smoke Exposure.” The conditions of SHS exposure were collected through the questionnaires filled out by women prepared for a baby within next 4–6 months. In our study, women who moved during the follow-up were excluded to avoid the bias induced by moving during perinatal period, which could ensure the stability of pre-natal living environment. Additionally, When couples are prepared for pregnancy, lifestyle and metabolic control before and during pregnancy would be maintained properly for a successful pregnancy (25). So the conditions of SHS exposure during prepared pregnancy period could be regarded as pregnancy period.

### Statistical Analysis

All the enrolled pregnant women in NFPHEP needed to be screened for the eligibility of inclusion into our study. The definition of loss to follow-up was that participants got preconception examination but had not received pre-natal or post-natal examination and questionnaires yet by 1 month after the expected date of confinement. To avoid the bias induced by moving during pregnancy, women who moved during the follow-up were excluded. The pregnant women who had missing data of PM_2.5_ concentration, birth weight, SHS conditions were excluded from the analysis. Extreme observations in other variables were replaced with missing data and were included in our analysis. Multiple imputations were also done for missing data, including the concentration of PM_2.5_ in different pregnancies, birth weight, and SHS conditions to conduct a sensitivity analysis.

The establishment of models was based on multivariable logistic regression, such as main effect item, interaction effect item, and other confounding factor adjustments. Four models were constructed on the basis of directed acyclic graphs (DAGs; Supplementary Figure A.2). Model 1 included PM_2.5_ concentration, different exposure levels of SHS, and the interaction between PM_2.5_ and SHS. Based on Model 1, maternal age at delivery and pre-pregnancy BMI were added in Model 2, and then neonate's sex (male or female), prolonged pregnancy ≥ 42 weeks (yes or no), and multiparity (yes or no) were added in the Model 3. Based on Model 3, preconception diabetes (yes or no), preconception hypertension (yes or no), family history of diabetes (yes or no), the highest maternal educational level (junior high school, senior high school, or college), the season of delivery (spring: March to May, summer: June to August, autumn: September to December, winter: November to February), and alcohol consumption status (current, former, or never drinker) were added in the fully adjusted Model 4. The statistical significance of interaction items was evaluated using the hypothesis test for the coefficient in the model. According to the DAGs, the major confounding factors were age at delivery and pre-pregnancy BMI, which were also confounding factors that need to be corrected most in the models. So the Model 2, which adjusted age at delivery and pre-pregnancy BMI, could be regarded as the primary results of models. Models 3 and 4 played supported and assistant roles in the study. The strategies of the confounding factors adjustment for the first-, second-, and third trimesters of pregnancy followed the full-adjusted principle, and all the three trimesters adopted Model 4 above to evaluate the trimester-specific interaction effect. Odds ratios (ORs) and their 95% CI were reported.

To further confirm the results of the interaction models, we conducted subgroup analyses stratified by SHS exposure (None exposure, Occasional exposure, and Frequent exposure). In each exposure level of SHS, binary logistic regression was established to evaluate the association between trimester-specific PM_2.5_ and fetal macrosomia. ORs and their 95% CI were reported.

The data cleaning process was conducted using Stata 16. All the interaction models and SHS subgroup analyses were performed using R 4.0.3, and “rms,” “mice,” “MASS,” “Rcpp,” “foreign,” “pool,” “maps,” “ggplot2” packages were used in analyses. A two-sided *p*-value < 0.05 was considered statistically significant.

## Results

**Figure 2:**
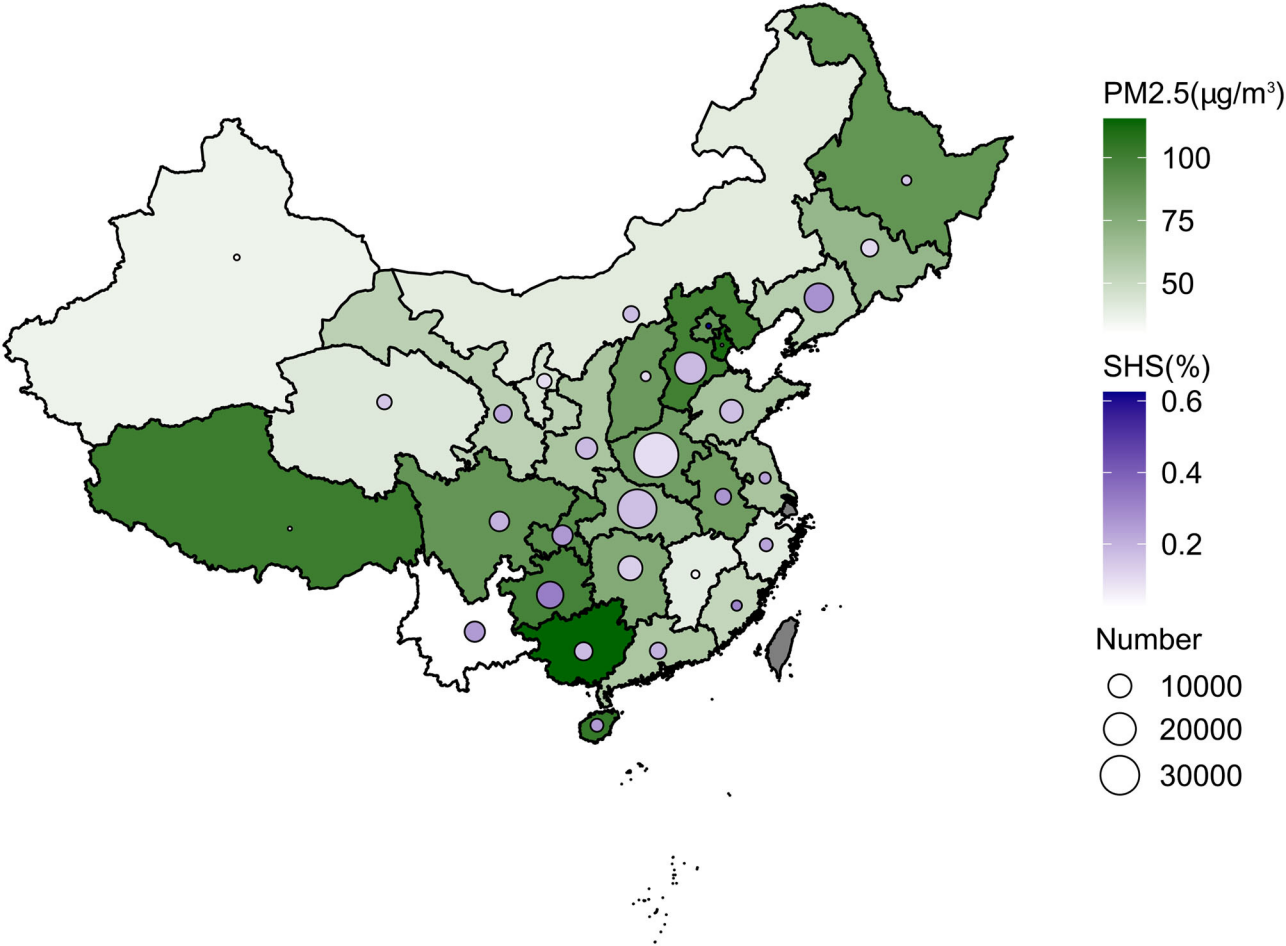
Distribution of PM_2.5_ concentration, SHS exposure, and a number of included pregnant women of study in whole pregnancy in province-level regions in mainland China.

Table 1 shows the baseline characteristic of women and neonates included in the study. Among all newborns (*n* = 197,877), macrosomia (*n* = 15,348) accounted for 7.76% and the last were non-macrosomia (*n* = 182,529). Male neonates (*n* = 9,191) were more than female neonates (*n* = 6,140) among the macrosomia group. The average birth weight of newborns in the whole study was 3,318.59 g. Other characteristics are also clearly shown in Table 1. The distributions of the sample size of included pregnant women, PM_2.5_ concentration, and SHS exposure in 220 counties are shown in maps in Figure 2.

**Table 1:** Baseline characteristics of the included pregnant women and neonates.

**Characteristics**	**Macrosomia**		**Overall**
	**Yes (*n* = 15,348)**	**No (*n* = 182,529)**	**(*n* = 197,877)**
Birth weight, g	4322.43 ± 469.90	3234.18 ± 428.42	3318.59 ± 520.74
Neonate's sex			
Male	9,191 (59.90%)	95,332 (52.20%)	104,523 (52.80%)
Female	6,140 (40.00%)	87,101 (47.70%)	93,241 (47.10%)
Missing	17 (0.10%)	96 (0.10%)	113 (0.10%)
Age at delivery, y	25.32 ± 3.97	25.23 ± 3.93	25.24 ± 3.94
Pre-pregnancy BMI^a^, kg/m^2^	21.30 ± 2.76	21.02 ± 2.60	21.04 ± 2.61
Prolonged pregnancy			
Yes	523 (3.40%)	4 103 (2.20%)	4 626 (2.30%)
No	14,825 (96.60%)	178,426 (97.80%)	193,251 (97.70%)
Multiparity			
Yes	11,894 (77.50%)	37,983 (20.80%)	49,877 (25.20%)
No	3,454 (22.50%)	144,546 (79.20%)	148,000 (74.80%)
Highest education level			
Junior high school	10,753 (70.10%)	128,280 (70.30%)	139,033 (70.30%)
Senior high school	2,892 (18.80%)	34,594 (19.00%)	37,486 (18.90%)
College	1,532 (10.00%)	17,368 (9.50%)	18,900 (9.60%)
Missing	171 (1.10%)	2,287 (1.20%)	2,458 (1.20%)
Pre-pregnancy diabetes mellitus			
Yes	3 (0.02%)	18 (0.05%)	21 (0.01%)
No	15,345 (99.98%)	178,426 (97.75%)	193,771 (97.93%)
Missing	0 (0.00%)	4,085 (2.20%)	4,085 (2.06%)
Pre-pregnancy hypertension			
Yes	8 (0.05%)	93 (0.10%)	101 (0.10%)
No	15,340 (99.95%)	182,436 (99.90%)	197,776 (99.90%)
Family history of diabetes mellitus			
Yes	100 (0.70%)	1,040 (0.60%)	1,140 (0.60%)
No	15,248 (99.30%)	181,489 (99.40%)	196,737 (99.40%)
Drinking during pregnancy			
No, has never drunk	15,205 (99.10%)	180,573 (98.90%)	195,778(98.90%)
No, but used to drink	99 (0.60%)	1,169 (0.60%)	1,268 (0.60%)
Yes	35 (0.20%)	720 (0.40%)	755 (0.40%)
Missing	9 (0.10%)	67 (0.10%)	76 (0.10%)
Season of delivery			
Spring	4,065 (26.50%)	48,959 (26.80%)	53,024 (26.80%)
Summer	2,421 (15.80%)	28,626 (15.70%)	31,047 (15.70%)
Autumn	3,897 (25.40%)	47,184 (25.90%)	51,081 (25.80%)
Winter	4,965 (32.30%)	57,760 (31.60%)	62,725 (31.70%)
Secondhand Smoking			
None exposure	12,320 (80.30%)	149,308 (81.80%)	161,628 (81.70%)
Occasional exposure	2,683 (17.50%)	29,845 (16.40%)	32,528 (16.40%)
Frequent exposure	345 (2.20%)	3,376 (1.80%)	3,721 (1.90%)

a *BMI, body mass index*.

Table 2 summarizes the mean concentration of PM_2.5_ during pregnancy according to different pregnancy stages. The whole, first-, second-, and third-semester pregnancy had 77.57, 73.35, 73.82, and 83.88 μg/m^3^ PM_2.5_ exposure in the macrosomia group, and 75.28, 71.01, 71.83, and 81.43 μg/m^3^ PM_2.5_ in the overall group.

**Table 2:** Mean PM_2.5_ concentration during pregnancy (μg/m^3^).

**Stage of pregnancy**	**Macrosomia**		**Overall (*n* = 197,877)**
	**Yes (*n* = 15,348)**	**No (*n* = 182,529)**	
First trimester	73.35 ± 29.25	70.81 ± 29.26	71.01 ± 29.27
Second trimester	73.82 ± 29.00	71.67 ± 29.97	71.83 ± 29.90
Third trimester	83.88 ± 32.46	81.23 ± 33.08	81.43 ± 33.04
whole pregnancy	77.57 ± 22.11	75.08 ± 22.93	75.28 ± 22.87

*PM_2.5_, Fine particulate matter*.

Table 3 shows the interaction between PM_2.5_ exposure in whole pregnancy and SHS exposure on risk of macrosomia. Among the four models, the latter model in turn added some confounding factors based on the previous model. In each interactive model, maternal exposure to PM_2.5_ during pregnancy was associated with an increased risk of macrosomia in whole pregnancy separately (*p* < 0.001; *p* < 0.001; *p* < 0.001; *p* < 0.001). However, there was no significant association between different frequencies of SHS exposure and risk of macrosomia, not only occasional SHS exposure (*p* = 0.246; *p* = 0.124; *p* = 0.116; *p* = 0.099) but also frequent SHS exposure (*p* = 0.374; *p* = 0.289; p = 0.289; *p* = 0.272). The interactive effect was statistically significant between maternal exposure to PM_2.5_ and SHS on the risk of macrosomia (interaction *p* < 0.05). In four different confounding factor-adjusted models, different frequencies of SHS exposure significantly enhanced the risk of macrosomia among pregnant women exposed to PM_2.5_ air pollutants (interaction *p* < 0.05). From Models 1–4, interactive effects between occasional SHS exposure and PM_2.5_ were significant (interaction *p* = 0.021; *p* = 0.010; *p* = 0.010; *p* = 0.010), and interaction effects between frequent SHS exposure and PM_2.5_ were also significant (interaction *p* = 0.050; *p* = 0.046; *p* = 0.051; *p* = 0.047). The higher frequency of SHS exposure prompted a stronger interaction between the two air pollutants. The ORs of all confounding factors in Model 4 are listed in Supplementary Table A.1. The interactions between PM_2.5_ exposure in whole pregnancy and SHS exposure on risk of macrosomia after multiple imputations are added in Supplementary Tables A.2, A.3.

**Table 3:** The interaction between PM_2.5_ exposure (10 μg/m^3^ increase) in whole pregnancy and SHS exposure on risk of macrosomia.

	**Odds ratio (95%CI)**	***p*-value**
	**Whole pregnancy**	
Model 1^a^		
PM_2.5_ exposure	1.041 (1.030,1.051)	<0.001
Occasional SHS^e^ exposure	0.416 (0.093,1.825)	0.246
Frequent SHS exposure	0.171 (0.003,7.798)	0.374
PM_2.5_ & Occasional SHS exposure^f^	1.020 (1.000,1.041)	0.021
PM_2.5_ & Frequent SHS exposure^g^	1.041 (1.000,1.094)	0.050
Model 2^b^		
PM_2.5_ exposure	1.041 (1.030,1.051)	<0.001
Occasional SHS exposure	0.308 (0.069,1.370)	0.124
Frequent SHS exposure	0.120 (0.002,5.647)	0.289
PM_2.5_ & Occasional SHS exposure	1.020 (1.000,1.041)	0.010
PM_2.5_ & Frequent SHS exposure	1.051 (1.000,1.094)	0.046
Model 3^c^		
PM_2.5_ exposure	1.041 (1.030,1.041)	<0.001
Occasional SHS exposure	0.308 (0.067,1.331)	0.116
Frequent SHS exposure	0.120 (0.002,5.695)	0.289
PM_2.5_ & Occasional SHS exposure	1.020 (1.010,1.041)	0.010
PM_2.5_ & Frequent SHS exposure	1.041 (1.000,1.094)	0.051
Model 4^d^		
PM_2.5_ exposure	1.041 (1.030,1.051)	<0.001
Occasional SHS exposure	0.282 (0.062,1.268)	0.099
Frequent SHS exposure	0.125 (0.002,5.278)	0.272
PM_2.5_ & Occasional SHS exposure	1.020 (1.010,1.041)	0.010
PM_2.5_ & Frequent SHS exposure	1.051 (1.000,1.094)	0.047

a *Unadjusted*.

b *Adjusted for age at delivery, pre-pregnancy BMI*.

c *Adjusted for age at delivery, pre-pregnancy BMI, neonatal sex, prolonged pregnancy, and multiparity*.

d *Adjusted for age at delivery, pre-pregnancy BMI, neonatal sex, prolonged pregnancy, multiparity, pre-pregnancy diabetes mellitus, pre-pregnancy hypertension, family history of diabetes mellitus, educational level, drinking during pregnancy, and season of delivery*.

e *SHS, secondhand smoking*.

f *The interaction between mean PM_2.5_ concentration of whole pregnancy and occasional SHS exposure*.

g *The interaction between mean PM_2.5_ concentration of whole pregnancy and frequent SHS exposure*.

Table 4 shows the interaction between PM_2.5_ exposure in the first trimester, second trimester, and third trimester of pregnancy and SHS exposure on risk of macrosomia. Each interactive model was based on full confounding factor-adjusted model. In the first trimester, the interaction effect was statistically significant between maternal exposure to PM_2.5_ and occasional SHS exposure on the risk of macrosomia (interaction *p* < 0.001). Occasionally exposed to SHS significantly enhanced the risk of macrosomia among pregnant women exposed to PM_2.5_ air pollutants (interaction *p* < 0.001). Frequently exposed to SHS was not associated with the risk of macrosomia among pregnant women exposed to PM_2.5_ air pollutants (interaction *p* = 0.100). In the second or third trimester, there was no interaction between maternal PM_2.5_ exposure and occasional exposure to SHS (interaction *p* = 0.081; *p* = 0.592). There was no interaction between maternal PM_2.5_ exposure and frequently exposed to SHS during the second or third trimester of pregnancy (interaction *p* = 0.302; *p* = 0.067). The interaction between PM_2.5_ exposure in the first trimester, second trimester, and third trimester of pregnancy and SHS exposure on risk of macrosomia after multiple imputations are added in Supplementary Table A.4.

**Table 4:** The interaction between PM_2.5_ exposure (10 μg/m^3^ increase) in the first-, second-, and third trimesters of pregnancy and SHS exposure on risk of macrosomia.

	**Odds ratio (95%CI)**	***p*-value**
First trimester^a^		
PM_2.5_ exposure	1.030 (1.020,1.041)	<0.001
Occasional SHS^e^ exposure^b^	0.148 (0.045,0.479)	0.001
Frequent SHS exposure	0.479 (0.022,9.539)	0.631
PM_2.5_ & Occasional SHS exposure	1.030 (1.020,1.051)	0.000
PM_2.5_ & Frequent SHS exposure	1.030 (0.990,1.072)	0.100
Second trimester^a^		
PM_2.5_ exposure	1.030 (1.020,1.041)	<0.001
Occasional SHS exposure^c^	0.737 (0.235,2.303)	0.604
Frequent SHS exposure	1.138 (0.051,23.807)	0.936
PM_2.5_ & Occasional SHS exposure	1.010 (0.990,1.030)	0.081
PM_2.5_ & Frequent SHS exposure	1.020 (0.980,1.051)	0.302
Third trimester^a^		
PM_2.5_ exposure	1.030 (1.020,1.030)	<0.001
Occasional SHS exposure^d^	1.424 (0.444,4.527)	0.551
Frequent SHS exposure	0.369 (0.017,7.548)	0.524
PM_2.5_ & Occasional SHS exposure	1.030 (0.990,1.020)	0.592
PM_2.5_ & Frequent SHS exposure	1.030 (0.990,1.062)	0.067

a *Adjusted for age at delivery, pre-pregnancy BMI, neonatal sex, prolonged pregnancy, multiparity, pre-pregnancy diabetes mellitus, pre-pregnancy hypertension, family history of diabetes mellitus, educational level, drinking during pregnancy, and season of delivery*.

b *The interaction between mean PM_2.5_ concentration of the first-trimester pregnancy and occasional SHS exposure*.

c *The interaction between mean PM_2.5_ concentration of the second-trimester pregnancy and occasional SHS exposure*.

d *The interaction between mean PM_2.5_ concentration of the third-trimester pregnancy and occasional SHS exposure*.

e *SHS, secondhand smoking*.

Table 5 and Supplementary Figure A.3 represent the association between PM_2.5_ concentration in different stages of pregnancy and the risk of macrosomia in different SHS Subgroups. The results of the whole pregnancy were consistent with the results of the first trimester. The higher exposure concentration of PM_2.5_ during the whole pregnancy or the first trimester among pregnant women was associated with the higher risk of macrosomia (OR = 1.041, *p* < 0.001; OR = 1.030, *p* < 0.001). Different frequencies of SHS exposure significantly increased the risk of macrosomia among pregnant women exposed to PM_2.5_ air pollutants, not only exposed to SHS occasionally (interaction *p* < 0.001; *p* < 0.001), but also exposed to SHS frequently (interaction *p* < 0.001; *p* < 0.001). Higher frequency of SHS exposure among pregnant women induced a stronger interactive effect between PM_2.5_ and SHS air pollutants during the whole pregnancy (Frequent exposure OR = 1.072 > Occasional exposure OR = 1.062) and the first trimester (Frequent exposure OR = 1.083 > Occasional exposure OR = 1.062). In the second and the third trimesters, maternal PM_2.5_ exposure was associated with an increased risk of macrosomia (OR = 1.030, *p* < 0.001; OR = 1.020, *p* < 0.001). But there was no interaction between maternal PM_2.5_ exposure and SHS exposure in the second (OR = 1.030, *p* < 0.001; OR = 1.051, *p* < 0.001; OR = 1.041, *p* = 0.069) and the third trimester of pregnancy(OR = 1.020, *p* < 0.001; OR = 1.051, *p* < 0.001; OR = 1.051, *p* = 0.007). Associations between PM_2.5_ concentration in different stages of pregnancy and the risk of macrosomia in different SHS subgroups after multiple imputations are added in Supplementary Table A.5.

**Table 5:** Association between PM_2.5_ concentration (10 μg/m^3^ increase) in different stages of pregnancy and the risk of macrosomia in different SHS Subgroups.

	**SHS^**b**^ subgroup**	**Odds ratio (95%CI)**	***p*-value**
Whole pregnancy^a^	None exposure	1.041 (1.030, 1.051)	<0.001
	Occasional exposure	1.062 (1.051, 1.083)	<0.001
	Frequent exposure	1.072 (1.030, 1.127)	0.001
First trimester^a^	None exposure	1.030 (1.020, 1.041)	<0.001
	Occasional exposure	1.062 (1.051, 1.083)	<0.001
	Frequent exposure	1.083 (1.041, 1.127)	<0.001
Second trimester^a^	None exposure	1.030 (1.020, 1.030)	<0.001
	Occasional exposure	1.051 (1.030, 1.062)	<0.001
	Frequent exposure	1.041 (1.000, 1.083)	0.069
Third trimester^a^	None exposure	1.020 (1.020, 1.030)	<0.001
	Occasional exposure	1.051 (1.030, 1.062)	<0.001
	Frequent exposure	1.051 (1.010, 1.094)	0.007

a *Adjusted for age at delivery, pre-pregnancy BMI, neonatal sex, prolonged pregnancy, multiparity, pre-pregnancy diabetes mellitus, pre-pregnancy hypertension, family history of diabetes mellitus, educational level, drinking during pregnancy, and season of delivery*.

b *SHS, secondhand smoking*.

## Discussion

In the full-adjusted interaction model, we found that maternal exposure to PM_2.5_ was associated with an increased risk of macrosomia in the whole pregnancy, the first-, second-, and third trimesters of pregnancy. In whole pregnancy, for every 10 μg/m^3^ increase in the concentration of PM_2.5_, the risk of macrosomia was increased by 4.1% among pregnant women. The interaction effect was statistically significant between maternal exposure to PM_2.5_ and SHS on the risk of macrosomia in the whole pregnancy and the first-trimester pregnancy. The higher frequency of SHS exposure prompted a stronger interaction between the two air pollutants in the whole pregnancy and the first-trimester pregnancy. In conclusion, in the whole and the first-trimester pregnancy, maternal exposure to SHS during pregnancy enhanced the risk of macrosomia among pregnant women who were exposed to PM_2.5_ air pollutants, and the interaction became stronger with the higher frequency of SHS exposure.

There is a rigorous methodology in our study. First, the process of data cleaning was conducted following strict screening standards. All the enrolled pregnant women in NFPHEP needed to be screened for the eligibility of inclusion into our study. Multiple imputations were added to process missing data. Second, the confounding factors were adjusted gradually in different models to avoid bias to the outcomes. Third, the subgroup analyses stratified by SHS exposure were conducted to further confirm the results of interactive models.

There are several innovations in our research. First, our study was conducted among a new study cohort, that is, the national pregnant Chinese women. Previous studies on the adverse effects of air pollution exposure on birth outcomes were mostly carried out in the developed countries or regions (26, 27). The differences between Chinese and people from other countries were reflected in several aspects, such as the higher exposure condition of maternal SHS (28, 29), higher PM_2.5_ pollution levels (30, 31), and more vulnerable metabolism characteristics of the Chinese population compared with people from other countries (32, 33). Hence, our study provided new evidence on the prognosis of air pollution-related diseases among the Chinese population, which represented 18.5% of the population of the world. Second, our study established a new interaction model between two human-caused air pollutants. Previous studies have focused on the interaction between genetic inheritance and environmental pollutants on birth outcomes (34, 35). No study has paid attention to the interaction between two air pollutants caused by human activities during different stages of pregnancy. Third, our study has identified a new risk that reinforced the association between maternal PM_2.5_ air pollutant exposure and macrosomia newborns. We found that maternal SHS exposure enhanced the risk of macrosomia associated with PM_2.5_ exposure.

Our study found that maternal exposure to PM_2.5_ during pregnancy was associated with an increased risk of macrosomia in whole pregnancy and different stages of pregnancy. The findings were consistent with a previous study on the association between PM_2.5_ exposure and birth weight (18). In CHEN et al.'s study (18), significant associations were found between the increased risk of macrosomia and every 10 μg/m^3^ increase of PM_2.5_ concentration over the first-, second-, and third trimesters in a nationwide prospective cohort study in China.

Our study suggested that there was an interaction between maternal exposure to SHS and PM_2.5_ air pollutants on the risk of macrosomia in the whole pregnancy and the first-trimester pregnancy. The higher frequency of SHS exposure prompted the stronger interaction between the two air pollutants in the whole pregnancy and the first-trimester pregnancy. No studies before have concentrated on the interaction effect of two air pollutants on adverse pregnancy outcomes, such as birth weight. The interaction between air pollutants and other factors on birth weight outcome has been reported in previous studies. Huang et al. (36) examined the interaction effects of pre-natally exposure to environmental tobacco smoke (ETS) and genotypes of cytochrome P4501A1 (CYP1A1), glutathione S-transferases (GSTs) on the risk of full-term low birth weight (FT-LBW). The study (36) revealed that gene polymorphisms of CYP1A1 and GSTs played modified roles in associations between pre-natal ETS exposure and FT-LBW. However, it was harder to take measures to change genetic endowments, compared with controlling manmade pollutant factors, such as PM_2.5_ and SHS exposure. Wang et al. (37) also assessed the interaction of air pollutants and meteorological factors on birth weight in Shenzhen, China. Wang et al. (37) found that, an interactive effect of air temperature and humidity on the relationship between PM10 exposure and SGA among newborns existed. Other studies also reported the interaction between air pollutants and life-related risk factors of mothers during pregnancy on fetal birth weight, such as maternal pre-pregnancy overweight (38), maternal employment (39), and maternal illicit drug use (40). However, in all, no studies before assessed the interaction effect of two air pollutants on birth weight outcomes.

The detailed mechanism of interaction between maternal exposure to SHS and PM_2.5_ air pollutants on the risk of macrosomia during pregnancy remained unclear. A few possible explanations or hypotheses were as follows. First, SHS is important indoor air pollution that could be inhaled into lung and blood circulation (12, 13, 41, 42). When SHS exposure from surrounding smokers and PM_2.5_ exposure from the atmosphere existed simultaneously, the concentration of PM_2.5_ changed and certain effects, such as synthesis, may happen. Hence, the interaction between maternal exposure to SHS and PM_2.5_ may exist. Secondly, studies on the association of PM_2.5_ air pollutants and newborn birth weight outcomes suggested that the changes of oxidative stress (43), immune response (44), and epigenetic regulation (45) may influence birth weight. Furthermore, studies on the association of maternal tobacco smoke exposure and fetal birth weight suggested that placental toxicity (46) and epigenetic modifications, such as DNA methylation (47), could be potential mediators affecting fetal birth weight outcomes. So epigenetic modification could be an explanation for the interaction between maternal exposure to SHS and PM_2.5_ air pollutants on the risk of macrosomia. Maybe the two air pollutants had some common gene methylation or overlapped molecular pathway of macrosomia leading to the interaction. In our study, SHS enhanced the risk of macrosomia among pregnant women who were exposed to PM_2.5_ air pollutants. The explanation maybe that the genes or signal pathways related to the association between SHS and macrosomia could be activated by the gene or signal pathway related to PM_2.5_. SHS exposure alone could not lead to macrosomia, because macrosomia-related genes were not activated by PM_2.5_. Our study suggested only in the whole pregnancy and the first trimester that the interaction between maternal exposure to SHS and PM_2.5_ air pollutants on the risk of macrosomia existed. The results indicated that exposure to PM_2.5_ in the first trimester had the biggest effect on birth weight. Maybe it was because that the exposure to air pollution in the first trimester could lead to placental adaptation through epigenetic modification (48).

Our study has some strengths and several potential limitations. The strengths included an emphasis on the harm of SHS to pregnant women, especially in high-exposed PM_2.5_ regions. Another strength was to point out that future studies could focus on the epigenetic aspects to explore the mechanism of birth weight changes associated with environmental pollutants. The study had also some limitations. The majority of pregnant women in NFPHEP were from rural areas in China, and thus, the conclusions made in this study were most pertinent to this region. Since the survey of SHS condition in our study was conducted through the questionnaire completed by the pregnant women, the collected information could be inevitably influenced by subjective ideas. Our research obtained the estimated PM_2.5_ concentration by the county-level data from satellite-retrieved aerosol optic depth rather than the ground-monitored levels, which may increase the exposure misclassification. This was also a limitation of our research. However, in China, the national air quality-monitoring network was established in 2013 such that PM_2.5_ measurements before 2013 were unavailable. Our study was conducted from January 1, 2010, to December 31, 2012, just before the training of the national air quality monitoring system. Additionally, the ground monitoring of PM_2.5_ was limited in most developing regions, while all participates enrolled in our project were from rural areas. So satellite-based PM_2.5_ prediction was common to be used to evaluate air quality at that time. In addition, our hindcast model showed higher accuracy than previous models because of the spatial clustering method and the long period of data used in model training. Although we used a satellite-based comprehensive model and assigned exposures according to country level, misclassification of the exposure could still exist. The pollution at micro-environmental levels (e.g., indoor, outdoor, or associated with commuting) or maternal activity patterns may have contributed to a misclassification. Although we ruled out women who moved during pregnancy in our study to avoid the misclassification of exposure, there still might be misclassification of the exposure that we could not control. In addition, wealth score, deprivation index, urban rurality, and other socioeconomic factors may have effects on birth weight and should be added to the models. Owing to the lack of such information in the original dataset, wealth score, deprivation index, urban rurality, and other socioeconomic factors are failed to be added into models as confounding factors. This was also one of the limitations of our research.

Generally, our study highlighted the impact of environmental air pollutants on adverse pregnancy outcomes, such as birth weight, and inspired us to emphasize controllable human activity-caused environmental pollutants, such as PM_2.5_ and SHS, which could be reduced or removed by taking some measures in the future. Our results strengthened the evidence that maternal exposure to PM_2.5_ during pregnancy was associated with an increased risk of macrosomia in China and meanwhile, firstly, suggested that SHS may enhance the risk of macrosomia associated with PM_2.5_. Therefore, pregnant women should avoid exposure to SHS during pregnancy, especially those who lived in areas with heavy PM_2.5_ pollution and who were in the first trimester of pregnancy. These findings helped to identify high-risk pregnant women for giving birth to macrosomia, providing an opportunity for targeted preventive interventions to protect the vulnerable populations as early as possible. Strengthening air pollution control could have a beneficial impact on avoiding adverse birth outcomes, improving the quality of the national population, and alleviating the national financial burden. Furthermore, the results of our study also suggested that the detailed mechanism of pregnancy outcome should focus more on epigenetic aspects of embryonic development in the future.

## Conclusion

In the whole pregnancy and the first-trimester pregnancy, maternal exposure to SHS during pregnancy enhanced the risk of macrosomia among pregnant women exposed to PM_2.5_ air pollutants, and the interaction became stronger with the higher frequency of SHS exposure.

## Data Availability Statement

The data analyzed in this study is subject to the following licenses/restrictions: Our research data were derived from the National Free Preconception Health Examination Project (NFPHEP). Requests to access these datasets should be directed to Hui Pan, PanHui@pumch.cn.

## Ethics Statement

The studies involving human participants were reviewed and approved by Institutional Review Board of the National Research Institute for Family Planning, Beijing, China. The patients/participants provided their written informed consent to participate in this study. Written informed consent was obtained from the individual(s) for the publication of any potentially identifiable images or data included in this article.

## Author Contributions

YL has drafted the work and completed the manuscript. YZ has made substantial contributions to the acquisition, analysis, or interpretation of data for the work. HP has provided editing and writing assistance for important intellectual content. SC has made substantial contributions to the conception or design of the work. All authors contributed to the article and approved the submitted version.

## Funding

This study was funded from the National Natural Science Foundation of China, grant/award number: 81673184; Beijing Municipal Natural Science Foundation, grant/award number: 7192153; CAMS Innovation Fund for Medical Science CAMS Initiative for Innovative Medicine, grant/award number: CIFMS-2016-I2M-1-008; the National Key Program of Clinical Science, grant/award number: WBYZ2011-873.

## Conflict of Interest

The authors declare that the research was conducted in the absence of any commercial or financial relationships that could be construed as a potential conflict of interest.

## Publisher's Note

All claims expressed in this article are solely those of the authors and do not necessarily represent those of their affiliated organizations, or those of the publisher, the editors and the reviewers. Any product that may be evaluated in this article, or claim that may be made by its manufacturer, is not guaranteed or endorsed by the publisher.
