# Supplementary material for: Maternal Secondhand Smoke Exposure Enhances Macrosomia Risk Among Pregnant Women Exposed to PM2.5: A New Interaction of Two Air Pollutants in a Nationwide Cohort
Source: Front Public Health. 2021 Nov 18;9:735699. doi: 10.3389/fpubh.2021.735699 (PMC8637054; doi:10.3389/fpubh.2021.735699)
Supplement: Supplementary file 1 [file Data_Sheet_1.PDF]

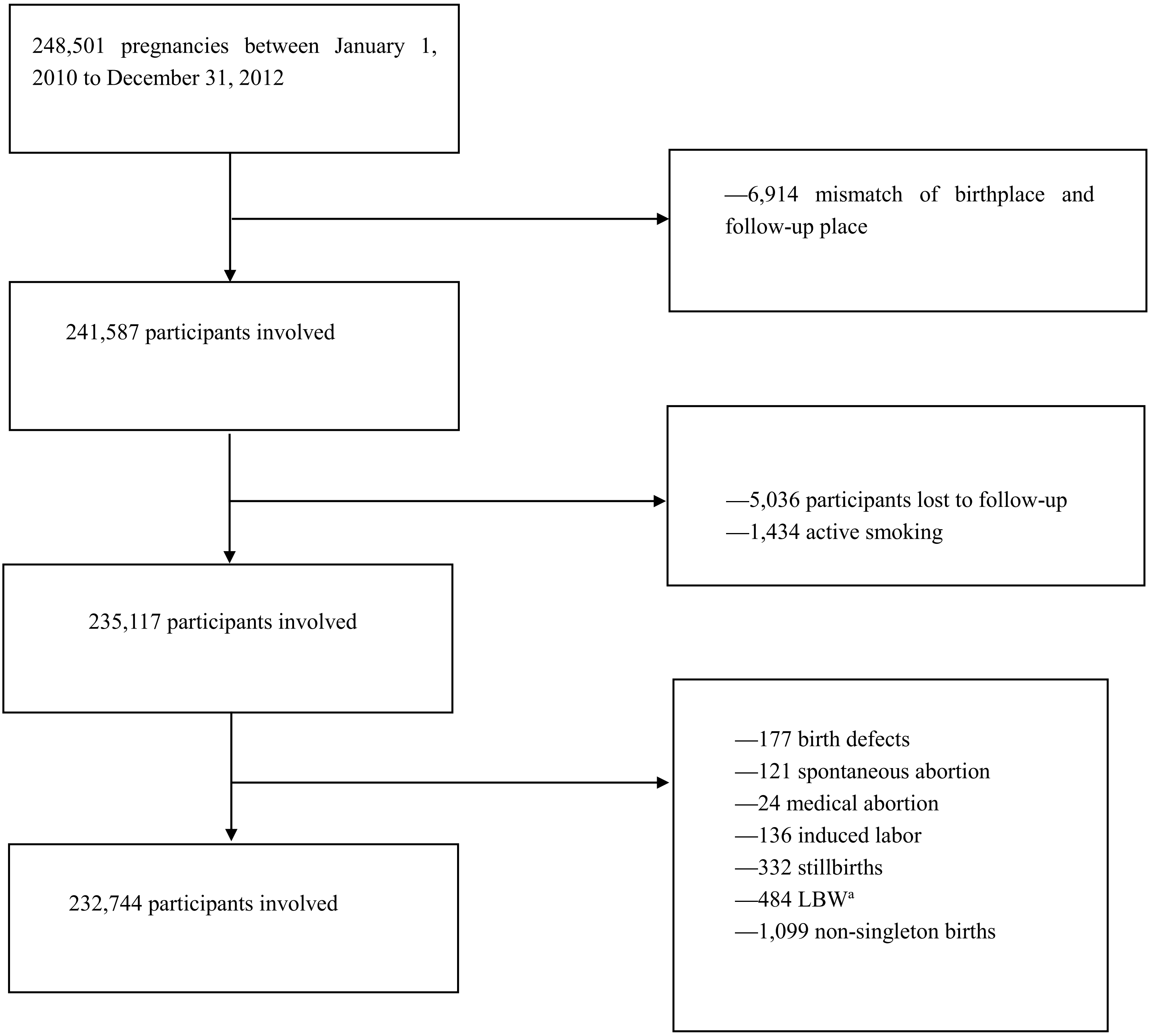

[a]LBW, low birth weight.

■ Figure A.1 Flowchart of participants inclusion and exclusion (after multiple imputations)

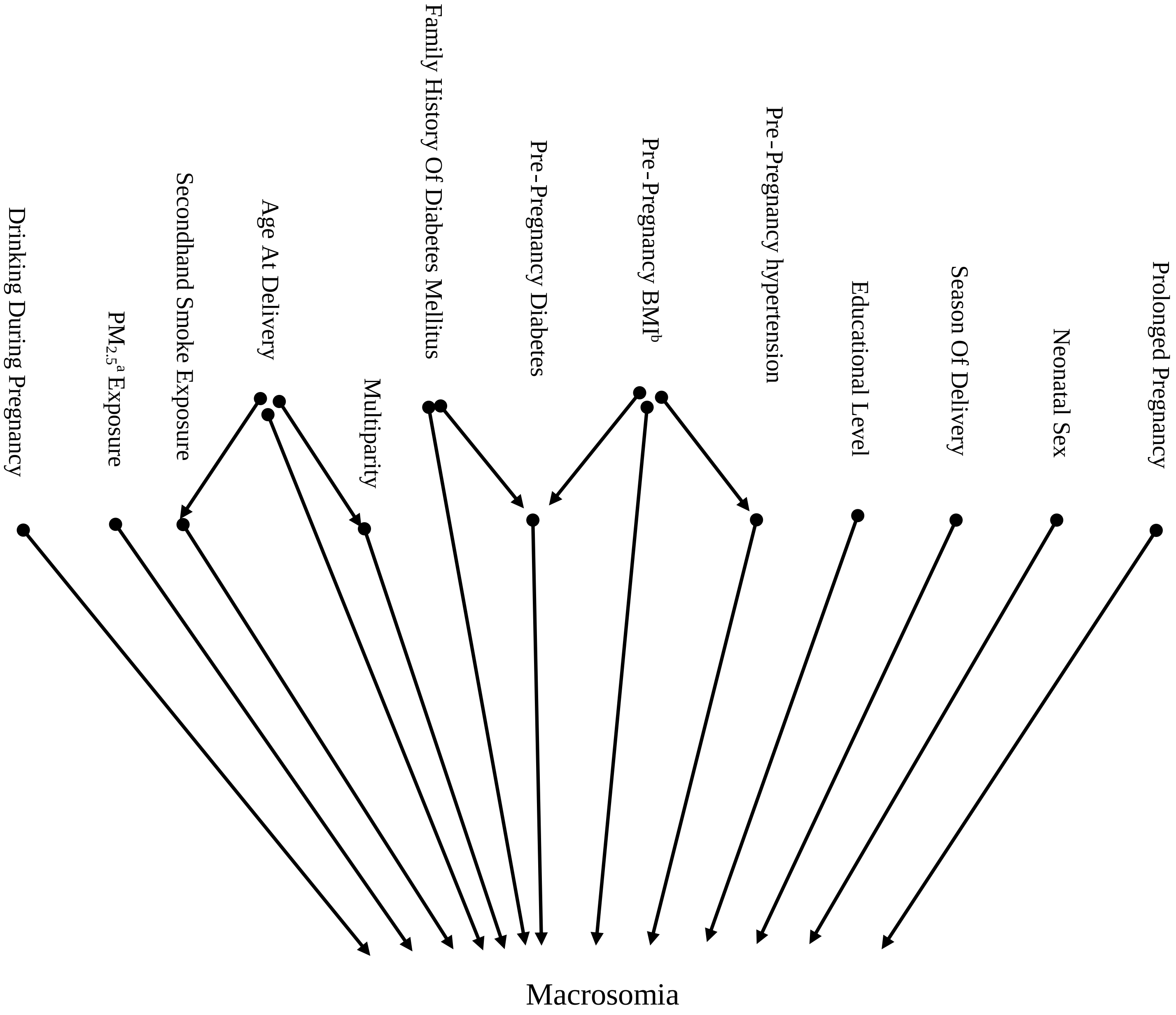

[a]$PM_{2.5}$, fine particulate matter.
[b]BMI, body mass index.

■ Figure A.2 DAGs (Directed Acyclic Graph) of models.

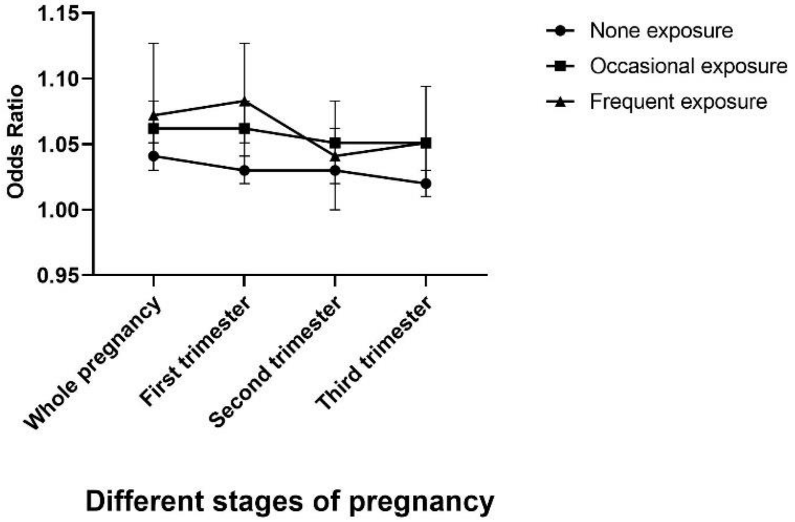

■ Figure A.3 Association between $PM_{2.5}$ concentration (10 μg/m^3 increase) in different stages of pregnancy and the risk of macrosomia in different Secondhand Smoke Subgroups.

■ TABLE A.1 The interaction between $PM_{2.5}$ exposure (10 μg/m^3 increase) in whole pregnancy and SHS exposure on risk of macrosomia.

	Odds Ratio(95%CI)	P value
	whole pregnancy [f]	
$PM_{2.5}$ [a] exposure	1.041(1.030,1.051)	<0.001
Occasional SHS [b] exposure	0.282(0.062,1.268)	0.098
Frequent SHS exposure	0.125(0.002,5.278)	0.272
Pre-pregnancy BMI[c]	1.411(1.331,1.495)	<0.001
Age at delivery	1.020(0.970,1.062)	0.477
Neonate's sex	0.043(0.031,0.060)	<0.001
Multiparity	1.808(1.172,2.764)	0.007
Prolonged pregnancy	53.21(20.558,134.853)	<0.001
Season of delivery (Spring)	reference	reference
Season of delivery (Summer)	1.424(0.834,2.411)	0.199
Season of delivery (Autumn)	1.629(1.010,2.594)	0.045
Season of delivery (Winter)	1.949(1.255,3.024)	0.003
Highest education level (Junior high school)	reference	reference
Highest education level (Senior high school)	1.538(1.000,2.389)	0.049
Highest education level (college)	2.865(1.613,5.016)	<0.001
Drinking during pregnancy (Never)	reference	reference
Drinking during pregnancy (Quit)	0.817(0.095,6.296)	0.852
Drinking during pregnancy (Still)	0.004(0.000,0.111)	0.002
Pre-pregnancy diabetes mellitus	105.234(0.000,2580866.058)	0.459
Pre-pregnancy hypertension	0.171(0.000,129.980)	0.637
Family history of diabetes mellitus	1.692(0.199,12.861)	0.623
$PM_{2.5}$ & Occasional SHS exposure [d]	1.020(1.010,1.041)	0.010
$PM_{2.5}$ & Frequent SHS exposure [e]	1.051(1.000,1.094)	0.047

[a]$PM_{2.5}$, fine particulate matter.
[b]SHS, secondhand smoking.
[c]BMI, body mass index.
[d]The interaction between mean $PM_{2.5}$ concentration of whole pregnancy and occasional SHS exposure
[e]The interaction between mean $PM_{2.5}$ concentration of whole pregnancy and frequent SHS exposure
[f]Adjusted for age at delivery, pre-pregnancy BMI, neonatal sex, prolonged pregnancy, multiparity, pre-pregnancy diabetes mellitus, pre-pregnancy hypertension, family history of diabetes mellitus, educational level, drinking during pregnancy, and season of delivery.

■ TABLE A.2 The interaction between $PM_{2.5}$ exposure (10 μg/m^3 increase) in whole pregnancy and SHS exposure on risk of macrosomia (after multiple imputations)

	Odds Ratio（95%CI）	P value
	whole pregnancy	
Model 1 [a]		
$PM_{2.5}$ [e] exposure	1.041(1.030,1.051)	<0.001
Occasional SHS [f] exposure	0.469(0.117,1.842)	0.281
Frequent SHS exposure	0.275(0.007,10.166)	0.491
$PM_{2.5}$ & Occasional SHS exposure [g]	1.020(1.000,1.037)	0.024
$PM_{2.5}$ & Frequent SHS exposure [h]	1.041(1.000,1.083)	0.063
Model 2 [b]		
$PM_{2.5}$ exposure	1.037(1.028,1.045)	<0.001
Occasional SHS exposure	0.464(0.117,1.827)	0.274
Frequent SHS exposure	0.259(0.006,9.562)	0.470
$PM_{2.5}$ & Occasional SHS exposure	1.019(1.000,1.036)	0.027
$PM_{2.5}$ & Frequent SHS exposure	1.039(0.996,1.084)	0.071
Model 3 [c]		
$PM_{2.5}$ exposure	1.036(1.027,1.044)	<0.001
Occasional SHS exposure	0.464(0.116,1.833)	0.275
Frequent SHS exposure	0.245(0.006,9.121)	0.453
$PM_{2.5}$ & Occasional SHS exposure	1.018(1.001,1.036)	0.031
$PM_{2.5}$ & Frequent SHS exposure	1.039(0.990,1.083)	0.076
Model 4 [d]		
$PM_{2.5}$ exposure	1.039(1.030,1.047)	<0.001
Occasional SHS exposure	0.453(0.113,1.796)	0.262
Frequent SHS exposure	0.255(0.006,9.577)	0.467
$PM_{2.5}$ & Occasional SHS exposure	1.018(1.000,1.036)	0.033
$PM_{2.5}$ & Frequent SHS exposure	1.038(0.995,1.083)	0.081

[a]Unadjusted

[b]Adjusted for age at delivery, pre-pregnancy BMI

[c]Adjusted for age at delivery, pre-pregnancy BMI, neonatal sex, prolonged pregnancy, and multiparity

[d]Adjusted for age at delivery, pre-pregnancy BMI, neonatal sex, prolonged pregnancy, multiparity, pre-pregnancy diabetes mellitus, pre-pregnancy hypertension, family history of diabetes mellitus, educational level, drinking during pregnancy, and season of delivery.

[e]$PM_{2.5}$, fine particulate matter.

[f]SHS, secondhand smoking.

[g]The interaction between mean $PM_{2.5}$ concentration of whole pregnancy and occasional SHS exposure

[h]The interaction between mean $PM_{2.5}$ concentration of whole pregnancy and frequent SHS exposure

■ TABLE A.3 The interaction between $PM_{2.5}$ exposure (10 μg/m^3 increase) in whole pregnancy and SHS exposure on risk of macrosomia (after multiple imputations)

	Odds Ratio(95%CI)	P value
	whole pregnancy [f]	
$PM_{2.5}$ [a] exposure	1.041(1.030,1.051)	<0.001
Occasional SHS [b] exposure	0.454(0.113,1.791)	0.262
Frequent SHS exposure	0.254(0.006,9.539)	0.467
Pre-pregnancy BMI[c]	1.357(1.280,1.438)	<0.001
Age at delivery	1.010(0.961,1.041)	0.768
Neonate's sex	0.044(0.032,0.059)	<0.001
Multiparity	1.808(1.195,2.714)	0.004
Prolonged pregnancy	62.862(25.785,149.568)	<0.001
Season of delivery (Spring)	reference	reference
Season of delivery (Summer)	1.357(0.825,2.240)	0.225
Season of delivery (Autumn)	1.629(1.051,2.524)	0.029
Season of delivery (Winter)	2.023(1.331,3.051)	0.001
Highest education level (Junior high school)	reference	reference
Highest education level (Senior high school)	1.305(0.860,1.931)	0.204
Highest education level (college)	2.023(1.195,3.395)	0.008
Drinking during pregnancy (Never)	reference	reference
Drinking during pregnancy (Quit)	1.138(0.151,7.548)	0.900
Drinking during pregnancy (Still)	0.006(0.000,0.123)	0.002
Pre-pregnancy diabetes mellitus	57.665(0.000,3149304.344)	0.517
Pre-pregnancy hypertension	0.092(0.000,66.703)	0.518
Family history of diabetes mellitus	1.774(0.224,12.663)	0.577
$PM_{2.5}$ & Occasional SHS exposure [d]	1.020(1.000,1.041)	0.033
$PM_{2.5}$ & Frequent SHS exposure [e]	1.041(1.000,1.072)	0.081

[a]$PM_{2.5}$, fine particulate matter.
[b]SHS, secondhand smoking.
[c]BMI, body mass index.
[d]The interaction between mean $PM_{2.5}$ concentration of whole pregnancy and occasional SHS exposure
[e]The interaction between mean $PM_{2.5}$ concentration of whole pregnancy and frequent SHS exposure
[f]Adjusted for age at delivery, pre-pregnancy BMI, neonatal sex, prolonged pregnancy, multiparity, pre-pregnancy diabetes mellitus, pre-pregnancy hypertension, family history of diabetes mellitus, educational level, drinking during pregnancy, and season of delivery.

■ TABLE A.4 The interaction between $PM_{2.5}$ exposure (10 μg/m^3 increase) in first trimester, second trimester and third trimester of pregnancy and SHS exposure on risk of macrosomia (after multiple imputations)

	Odds Ratio (95%CI)	P value
First trimester [a]		
$PM_{2.5}$[e] exposure	1.032(1.025,1.040)	<0.001
Occasional SHS [f] exposure [b]	0.235(0.079,0.692)	0.009
Frequent SHS exposure	0.665(0.037,11.229)	0.780
$PM_{2.5}$ & Occasional SHS exposure	1.028(1.014,1.042)	<0.001
$PM_{2.5}$ & Frequent SHS exposure	1.028(0.990,1.063)	0.105
Second trimester [a]		
$PM_{2.5}$ exposure	1.027(1.020,1.033)	<0.001
Occasional SHS exposure [c]	0.864(0.301,2.469)	0.786
Frequent SHS exposure	1.598(0.089,27.138)	0.748
$PM_{2.5}$ & Occasional SHS exposure	1.010(0.997,1.023)	0.104
$PM_{2.5}$ & Frequent SHS exposure	1.016(0.983,1.050)	0.327
Third trimester [a]		
$PM_{2.5}$ exposure	1.028(1.022,1.036)	<0.001
Occasional SHS exposure [d]	1.842(0.628,5.368)	0.264
Frequent SHS exposure	0.896(0.049,15.368)	0.941
$PM_{2.5}$ & Occasional SHS exposure	1.000(0.980,1.012)	0.916
$PM_{2.5}$ & Frequent SHS exposure	1.021(0.991,1.051)	0.160

[a]Adjusted for age at delivery, pre-pregnancy BMI, neonatal sex, prolonged pregnancy, multiparity, pre-pregnancy diabetes mellitus, pre-pregnancy hypertension, family history of diabetes mellitus, educational level, drinking during pregnancy, and season of delivery.

[b]The interaction between mean $PM_{2.5}$ concentration of the first trimester pregnancy and occasional SHS exposure.

[c]The interaction between mean $PM_{2.5}$ concentration of the second trimester pregnancy and occasional SHS exposure.

[d]The interaction between mean $PM_{2.5}$ concentration of the third trimester pregnancy and occasional SHS exposure.

[e]$PM_{2.5}$, fine particulate matter.

[f]SHS, secondhand smoking.

■ TABLE A.5 Association between $PM_{2.5}$ concentration (10 μg/m^3 increase) in different stages of pregnancy and the risk of macrosomia in different SHS Subgroups (after multiple imputations)

	SHS [b] Subgroup	Odds Ratio(95%CI)	P value
Whole pregnancy [a]	None exposure	1.038(1.030,1.051)	<0.001
	Occasional exposure	1.062(1.041,1.083)	<0.001
	Frequent exposure	1.066(1.020,1.116)	0.003
First trimester [a]	None exposure	1.030(1.020,1.041)	<0.001
	Occasional exposure	1.062(1.041,1.072)	<0.001
	Frequent exposure	1.083(1.041,1.127)	<0.001
Second trimester [a]	None exposure	1.026(1.019,1.034)	<0.001
	Occasional exposure	1.042(1.027,1.056)	<0.001
	Frequent exposure	1.032(0.995,1.071)	0.086
Third trimester [a]	None exposure	1.025(1.019,1.032)	<0.001
	Occasional exposure	1.043(1.029,1.056)	<0.001
	Frequent exposure	1.044(1.009,1.007)	0.013

[a]Adjusted for age at delivery, pre-pregnancy BMI, neonatal sex, prolonged pregnancy, multiparity, pre-pregnancy diabetes mellitus, pre-pregnancy hypertension, family history of diabetes mellitus, educational level, drinking during pregnancy, and season of delivery.

[b]SHS, secondhand smoking.
